# Disease-Associated Mutations of the STAT5B SH2 Domain Regulate Cytokine-Driven Enhancer Function and Mammary Development

**DOI:** 10.1007/s10911-025-09582-8

**Published:** 2025-03-31

**Authors:** Hye Kyung Lee, Jakub Jankowski, Chengyu Liu, Lothar Hennighausen

**Affiliations:** 1 Section of Genetics and Physiology, National Institute of Diabetes and Digestive and Kidney Diseases, US National Institutes of Health, Bethesda, MD 20892 USA; 2 Transgenic Core, National Heart, Lung, and Blood Institute, US National Institutes of Health, Bethesda, MD 20892 USA

## Abstract

Mammary gland development during pregnancy is controlled by lactogenic hormones via the JAK2-STAT5 pathway. Gene deletion studies in mice have revealed the crucial roles of both STAT5A and STAT5B in establishing the genetic programs necessary for the development of mammary epithelium and successful lactation. Several hundred single nucleotide polymorphisms (SNPs) have been identified in human STAT5B, although their pathophysiological significance remains largely unknown. The SH2 domain is vital for STAT5B activation, and this study focuses on the impact of two specific missense mutations identified in T cell leukemias, the substitution of tyrosine 665 with either phenylalanine (Y665F) or histidine (Y665H). By introducing these human mutations into the mouse genome, we uncovered distinct and opposite functions. Mice harboring the STAT5B^Y665H^ mutation failed to develop functional mammary tissue, resulting in lactation failure, while STAT5B^Y665F^ mice exhibited accelerated mammary development during pregnancy. Transcriptomic and epigenomic analyses identified STAT5B^Y665H^ as Loss-Of-Function (LOF) mutation, impairing enhancer establishment and alveolar differentiation, whereas STAT5B^Y665F^ acted as a Gain-Of-Function (GOF) mutation, elevating enhancer formation. Persistent hormonal stimulation through two pregnancies led to the establishment of enhancer structures, gene expression and successful lactation in STAT5B^Y665H^ mice. Lastly, we demonstrate that *Olah*, a gene known to drive life-threatening viral disease in humans, is regulated by STAT5B through a candidate four-partite super-enhancer. In conclusion, our findings underscore the role of human STAT5B variants in modulating mammary gland homeostasis and their critical impact on lactation.

**Supplementary Information:**

The online version contains supplementary material available at 10.1007/s10911-025-09582-8.

## Introduction

Transcription factors belonging to the Signal Transducers and Activators of Transcription (STAT) family respond to cytokines, initiating both common and cell-specific genetic programs [1]. STAT5, the original member of the STAT family, plays an essential role in cytokine signaling within the hematopoietic system [2], mammary gland [3–5], body growth [6–9], and liver metabolism [9], as evidenced and confirmed through experimental mouse genetics [2, 4]. Comprising two highly conserved variants, STAT5A [10, 11] and STAT5B [11–13], it mediates prolactin signaling during pregnancy, orchestrating the formation and expansion of mammary alveoli to facilitate extensive milk production during lactation [4, 14] essential for offspring sustenance. Specifically, STAT5A/B bind to precise genomic locations, so called GAS motifs (TTCnnnGAA), at mammary enhancers and super-enhancers, thereby triggering the induction of milk protein gene expression up to 10,000-fold, during pregnancy and lactation [14–19]. While investigations utilizing mouse genetics have shed light on the modulatory role of STAT5A in mammary alveolar differentiation [4], understanding the precise contribution of STAT5B in mammary gland development is unclear due to the infertility of *Stat5b*-null mice, preventing direct investigation.

Based on available databases, approximately one third of amino acids in the human *STAT5B* genes have acquired missense mutations, but genotype–phenotype correlation remains limited. Inactivating mutations in STAT5B, associated with Laron syndrome, have been documented in humans [8, 20, 21], while potential activating mutations have been observed in patients with T cell leukemias [21, 22]. Since STAT5A/B responds to cytokine stimuli, the biological consequences of missense mutations might be detected only under specific physiological conditions, such as lactation or a challenged immune system. Although informative, overexpression studies, both in cell lines and mice, may be of limited significance in shedding light on the physiological and pathophysiological functions of missense mutations. A more promising approach involves the introduction of human mutations into the mouse genome, thus permitting investigations on their impact on key target tissues and different physiological states. Development of mammary glands during pregnancy and differentiation during lactation are exquisitely dependent on defined levels of STAT5 [4, 5, 14], making this organ a perfect test system to investigate the biological impact of naturally occurring variants.

In this study, we examine the biological consequences of two distinct human STAT5B mutations that replace tyrosine 665 (Y665) with either phenylalanine (Y665F) or histidine (Y665H) [21, 22]. We have incorporated the STAT5B^Y665F^ and STAT5B^Y665H^ missense mutations into the mouse genome and employed lactation, an inherent physiological process in mammalian development, to demonstrate the effects of single amino acid alterations on genetic programs within a hormonally regulated signaling pathway, and how the organism adjusts to these changes. Furthermore, we explore how the activating and inactivating STAT5B missense mutations influence the function of its twin, STAT5A. This significant example exposes fundamental principles of naturally occurring variants fine tuning mammary gland physiology and lactation and the capacity of adaptation in response to modifications in transcription factor activity.

## Materials and Methods

### Mice

All animals were housed and handled according to the Guide for the Care and Use of Laboratory Animals (8th edition) and all animal experiments were approved by the Animal Care and Use Committee (ACUC) of National Institute of Diabetes and Digestive and Kidney Diseases (NIDDK, MD) and performed under the NIDDK animal protocol K089-LGP-20. CRISPR/Cas9 and base editing [23, 24] targeted mice were generated using C57BL/6 N mice (Charles River) by the Transgenic Core of the National Heart, Lung, and Blood Institute (NHLBI). Single-guide RNAs (sgRNA) were obtained using Thermo Fisher Scientific’s In Vitro Transcription Service (Supplementary Table 1). Single-strand oligonucleotide donor was obtained from IDT. A plasmid containing the adenine base editor (ABE 7.10 provided by Dr. David Liu’s laboratory) was used to synthesize ABE mRNA using Thermo Fisher’s mMESSAGE mMACHINE T7 In Vitro Transcription Kit. For the Y665H mutant mice, the ABE mRNA (50 ng/µl) and Y665H sgRNA (20 ng/µl) were co-microinjected into the cytoplasm of fertilized eggs collected from superovulated C57BL/6 N female mice (Charles River Laboratories). For the Y665F mutant mice, a single-strand oligonucleotide donor contained the desired Y (TAC) to F (TTT) change and a silent C to G change. The silent mutation does not result in amino acid change but can destroy the sgRNA PAM and hence stopping Cas9 from further cutting after the oligo template was successfully knocked in. The Y665F sgRNA was first mixed with Cas9 protein (IDT) to form Cas9 RNP complex, which was co-electroporated with the oligo template into zygotes collected from C57BL/6 N mice using a Nepa21 electroporator (Nepa Gene Co) following procedures described by Kenako [25]. The microinjected or electroporated zygotes were cultured overnight in M16 medium (Millipore Sigma) at 37^o^C with 6% CO_2_. Those embryos that reached 2-cells stage of development were implanted into the oviducts of pseudopregnant surrogate mothers (Swiss Webster mice from Charles River). All mice born to the foster mothers were genotyped by PCR amplification and Sanger sequencing (Quintara Biosciences) and automate genotyping using a TaqMan-based assay (TransnetYX) with genomic DNA from mouse tails.

Two-months old mice were used in the experiments, following the ARRIVE guidelines (https://arriveguidelines.org/). Wild-type littermate mice of heterozygous Y665H and Y665F mutant mice were used as controls. Tissues were collected from mice and used immediately or stored at -80 °C.

### Whole Exome Sequencing and Data Analysis

Genomic DNA was isolated from tail tissue using Wizard Genomic DNA Purification Kit (Promega). Exome sequencing and bioinformatics analyses were performed at Psomagen. Target capture for the exome was performed on each sample using SureSelect Mouse All Exon kit (Agilent Technologies). DNA was subjected to SureSelect Target Enrichment System for paired-end DNA library preparation. Whole exome sequencing was performed on a NovaSeq 6000 instrument (Illumina).

Sequencing reads were aligned to the mouse reference (mm10) using BWA (version 0.7.10). After excluding chimeric reads, the duplicated reads were eliminated using Picard. GATK3.v4 ‘IndelRealigner’ and ‘Table Recalibration’ were used for local realignment and for recalibrating the quality scores, respectively. For SNV/Indel calling in multi-sample analysis, GATK ‘HaplotypeCaller’ was used for comparison with the reference genome. For SNV calling in matched-pair analysis, ‘Selectvariants’ was used to compare the difference between WT and mutant mouse. Annotation for all variants was made using dbSNP142.

### RNA Isolation and Quantitative Real-Time PCR (qRT–PCR)

Total RNA was extracted from frozen mammary tissue of wild type and mutant mice using a homogenizer and the PureLink RNA Mini kit according to the manufacturer’s instructions (Thermo Fisher Scientific). The concentration and quality of RNA were assessed by an Agilent Bioanalyzer 2100 (Agilent Technologies, CA). Total RNA (1 µg) with more than RIN value 8.0 was reverse transcribed for 50 min at 50 °C using 50 µM oligo dT and 2 µl of SuperScript III (Thermo Fisher Scientific) in a 20 µl reaction. Quantitative real-time PCR (qRT-PCR) was performed using TaqMan probes (*Csn1s1*, Mm01160593_m1; *Csn2*, Mm04207885_m1; *Csn1s2a*, Mm00839343_m1; *Csn1s2b*, Mm00839674_m1; *Csn3*, Mm02581554_m1; *Wap*, Mm00839913_m1; *Cish*, Mm01230623_g1; mouse *Gapdh*, Mm99999915_g1, Thermo Fisher scientific) on the CFX384 Real-Time PCR Detection System (Bio-Rad) according to the manufacturer’s instructions. PCR conditions were 95 °C for 30s, followed by 40 cycles of 95 °C for 15s, and 60 °C for 30s. All reactions were done in triplicate and normalized to the housekeeping gene *Gapdh*. Relative differences in PCR results were calculated using the comparative cycle threshold (*C*_*T*_) method and normalized to *Gapdh* levels.

### Total RNA Sequencing (RNA-seq) and Data Analysis

Ribosomal RNA was removed from 1 mg of total RNAs with more than RIN value 8.0and cDNA was synthesized using SuperScript III (Invitrogen). Libraries for sequencing were prepared according to the manufacturer’s instructions with TruSeq Stranded Total RNA Library Prep Kit with Ribo-Zero Gold (Illumina, RS-122-2301) and paired-end sequencing was done with a NovaSeq 6000 instrument (Illumina).

Total RNA-seq read quality control (Supplementary Table 2) was done using Trimmomatic [26] (version 0.36) and STAR RNA-seq [27] (version 2.7.11b) using paired-end mode was used to align the reads (mm10). HTSeq (0.9.1) [28] was to retrieve the raw counts and subsequently, R (version 4.2.3) (https://www.R-project.org/), Bioconductor [29] and DESeq2 [30] were used. Additionally, the RUVSeq [31] package was applied to remove confounding factors. Data quality was assessed via PCA plot (Supplementary Table 2). The data were pre-filtered keeping only those genes, which have at least ten reads in total. Genes were categorized as significantly differentially expressed with log2 fold change > 1 or <-1 and adjusted p-value (pAdj) < 0.05 corrected for multiple testing using the Benjamini-Hochberg method were considered significant and then conducted gene enrichment analysis (GSEA, https://www.gsea-msigdb.org/gsea/index.jsp and Metascape, https://metascape.org/gp/index.html#/main/step1). The visualization was done using dplyr (https://CRAN.R-project.org/package=dplyr) and ggplot2 [32].

### Chromatin Immunoprecipitation Sequencing (ChIP-seq), Co-ChIP, and Data Analysis

The frozen-stored tissues were ground into powder in liquid nitrogen. Chromatin was fixed with 1% formaldehyde (Sigma-Aldrich) for 10 min at room temperature and then quenched with 0.125 M glycine for 10 min at room temperature. Nuclei were isolated using Farnham Lysis Buffer (5 mM PIPES pH 8.0, 85 mM KCl, 0.5% NP-40, supplemented with PMSF and proteinase inhibitor cocktails). The chromatin was fragmented to 250–500 bp using sonicator 3000 (30 cycles; 20 s pulse/20 s rest, Misonix Sonicators), checked on DNA gel, and further lysed in RIPA buffer. One milligram of total protein was immunoprecipitated with Dynabeads Protein A (Novex) coated with ChIP-seq grade antibodies: STAT5A (Santa Cruz Biotechnology, sc-271542), STAT5B (R&D systems, AF1584; ThermoFisher scientific, 13-5300), NFIB (Sigma-Aldrich, HPA003956), H3K27ac (Abcam, ab4729) and RNA polymerase II (Abcam, ab5408). Libraries for next-generation sequencing were prepared with NEBNext Ultra II DNA library prep kit for illumina (New England Biolabs, M0287L) and sequenced with the NovaSeq 6000 instrument (Illumina).

For the dimerization of STAT5A/B, chromatin fragments were selectively precipitated by STAT5A antibody, followed by elution facilitated by an IgG elution buffer, pH of 2.8 (Thermo Scientific), and subsequent neutralization employing 1 M Tris buffer, pH of 9.5 (Thermo Scientific). The resultant eluted chromatin underwent a secondary precipitation step using STAT5B antibody.

Quality filtering and alignment of the raw reads (Supplementary Table 2) was done using Trimmomatic [26] (version 0.36) and Bowtie [33] (version 1.3.1), with the parameter ‘-m 1’ to keep only uniquely mapped reads, using the reference genome mm10. Picard tools (version 2.9.2) (Broad Institute. Picard, http://broadinstitute.github.io/picard/. 2016) was used to remove duplicates and subsequently, Homer [34] (version 4.10.4) and deepTools [35] (version 3.5.4) software was applied to generate bedGraph files and normalize coverage, separately. Integrative Genomics Viewer [36] (version 2.3.98) was used for visualization. MACS2 [37] peak-finding algorithm was used to identify regions of ChIP-seq enrichment over the background, utilizing input files generated from our previous research to detect regulatory elements. For visualization, the total reads number of mapped result in each sample was normalized to 10 million and background signals of < 2 were eliminated. GAS motifs were searched with IGV browser (version 2.12.3). To assess antibody specificity and immunoprecipitation (IP) efficiency (Supplementary Table 2), we evaluated ChIP-seq peaks for mammary-specific, immune cell-specific, and common STAT downstream genes that are regulated via JAK/STAT signaling pathway. Each ChIP-seq experiment was conducted for two-three replicates and the correlation between the replicates was analyzed by Pearson and Spearman correlation using deepTools (Supplementary Table 2).

To identify the activity of super-enhancers in WT and mutant mice, we calculated the normalized read coverage of mammary-specific super-enhancer regions previously identified [15] using STAT5A, STAT5B, and H3K27ac ChIP-seq datasets. This analysis was performed utilizing computeMatrix from deepTools (version 3.1.3), included generating tag density plots.

### STAT5B Protein Structure

The monomeric structure of mouse STAT5B (Uniprot accession P42232) was obtained from the AlphaFold Protein Structure Database [38]. Two STAT5B monomer was aligned and then generated a dimer in the crystal structure template of the mouse STAT3 dimer (RCDB ID: 1bg1) [39] using PyMol (https://www.pymol.org/). Additionally, the DNA fragment was coordinated from 1bg1.

### Histology and Immunohistochemistry

Fourth inguinal mammary glands tissues from wild-type and *mutant mice* were collected at virgin and days 13 and 18 of pregnancy. Isolated mammary tissues were fixed with 10% neutral formalin solution and dehydrated in 70% EtOH. Samples were processed for paraffin sections and stained with hematoxylin and eosin by standard methods (Histoserv).

Tissue slides were rehydrated through a graded ethanol series (100%, 95%, 70%, and 50%) followed by immersion in water. Antigen retrieval was performed by boiling the slides in Antigen Unmasking Solution (Vector Laboratories) for 10 min. Endogenous peroxidase activity was quenched by incubating the slides in freshly prepared 3% hydrogen peroxide for 10 min. After rinsing in water, non-specific binding was blocked by incubating the slides with 2.5% goat serum (Vector Laboratories) for 1 h at room temperature. The expression of STAT5A and STAT5B proteins was detected by overnight incubation with primary antibodies: STAT5A (1:200, Santa Cruz Biotechnology, sc-271542) and STAT5B (1:200, R&D Systems, AF1584) at room temperature. The slides were subsequently incubated with the appropriate secondary antibody (Goat anti-mouse or anti-rabbit IgG, Vector Laboratories, MP-7452 and MP-7451) for 1 h. Detection was carried out using the DAB (3,3’-diaminobenzidine) substrate (Vector Laboratories) for 10 min. Following water washes, nuclear counterstaining was achieved by immersing the slides in hematoxylin solution (Sigma-Aldrich) for 3 min. The slides were then dehydrated through an ascending ethanol series (50%, 70%, 95%, 100%) and cleared in xylene. Finally, the slides were mounted using Permount (Fischer Chemical), and images were captured using Keyence BZ-9000 microscope.

### Whole Mount Staining

For whole-mount analysis, an entire mammary gland was flattened on microscopic slides, fixed overnight in Carnoy’s solution (ethanol 6: chloroform 3:acetic acid 1), washed in 70%, 50%, 30%, 10% ethanol and distilled water for 15 min, respectively, and then stained overnight in carmine alum to visualize the ductal trees and alveolar buds. After washing in 70%, 90%, 95%, 100% ethanol, tissues were cleared and stored in xylene. Finally, the slides were mounted using Permount (Fischer Chemical) assessed using Keyence BZ-9000 microscope.

### Western Blot

Total T cells from isolated splenocytes were isolated using EasySep Mouse T cell isolation kit (Stemcell, #19851) and cultured with recombinant mouse IL-2 and IL-7 (R&D systems) for 24 h. Proteins (100 mg) were extracted with lysis buffer (50 mM Tris-Cl pH 8.0, 150 mM NaCl, 0.5% Na-DOC, 1% NP-40, 0.1% SDS, 5 mM EDTA, 1 mM PMSF, and protease inhibitor cocktail), separated on a 4–12% NuPage gradient gel (Invitrogen) and transferred to a PVDF membrane (Invitrogen). Membranes were blocked for 1 h with 5% nonfat dry milk in PBS-T buffer (PBS containing 0.1% Tween 20) and incubated for 1.5 h at 4 °C with the primary antibody against STAT5B (ThermoFisher scientific, 13-5300), phosphor-STAT5 (Abcam, ab32364) and GAPDH (Santa cruz, sc-47724). After washing, membranes were incubated for 1 h with HRP-conjugated secondary antibodies (Cell signaling). Labeled protein bands were detected using an enhanced chemiluminescence system (Thermo scientific) and Amersham Imager 600 (GE healthcare).

### Flow Cytometry

Blood was collected from retro-orbital sinus into Eppendorf tubes in the presence of 5 mM ethylenediaminetetraacetic acid (EDTA, Sigma). After cell surface and fixable viability staining of single-cell suspensions with fluorophore-conjugated antibodies in PBS, intracellular staining was performed after fixing and permeabilizing cells with the Foxp3/Transcription Factor Staining kit (eBioscience) per manufacturer’s instructions. The following antibodies were used for flow cytometry: CD4, CD8, CD45R (Biolegend), 7AAD, annexin V (BD Biosciences). Stained cells were acquired using BD FACSCanto II and BD LSR Fortessa flow cytometry operated by 5-laser Cytek Aurora performed with FlowJo v10.

### Statistical Analyses

All samples that were used for qRT-PCR and RNA-seq were randomly selected, and blinding was not applied. For comparison of samples, data were presented as standard deviation in each group and were evaluated with a 1-way or 2-way ANOVA multiple comparisons using PRISM GraphPad (version 10.1.1). Statistical significance was obtained by comparing the measures from wild-type or control group, and each mutant group. A value of *p < 0.05, **p < 0.001, ***p < 0.0001, ****p < 0.00001 was considered statistically significant. ns, no significant. The relationship between percentage of offspring in each genotype and the expected Mendelian ratios was statistically tested using a Chi-square test (https://www.standarddeviationcalculator.io/chi-square-calculator).

### Data Availability

The ChIP-seq and RNA-seq generated in this study have been deposited in the Gene Expression Omnibus (GEO) database under accession code GSE270652 and GSE270656. Other data were obtained from GEO database under accession code GSE115370, GSE145193, GSE161620, GSE115370 and GSE231441.

## Results

**Fig. 1:**
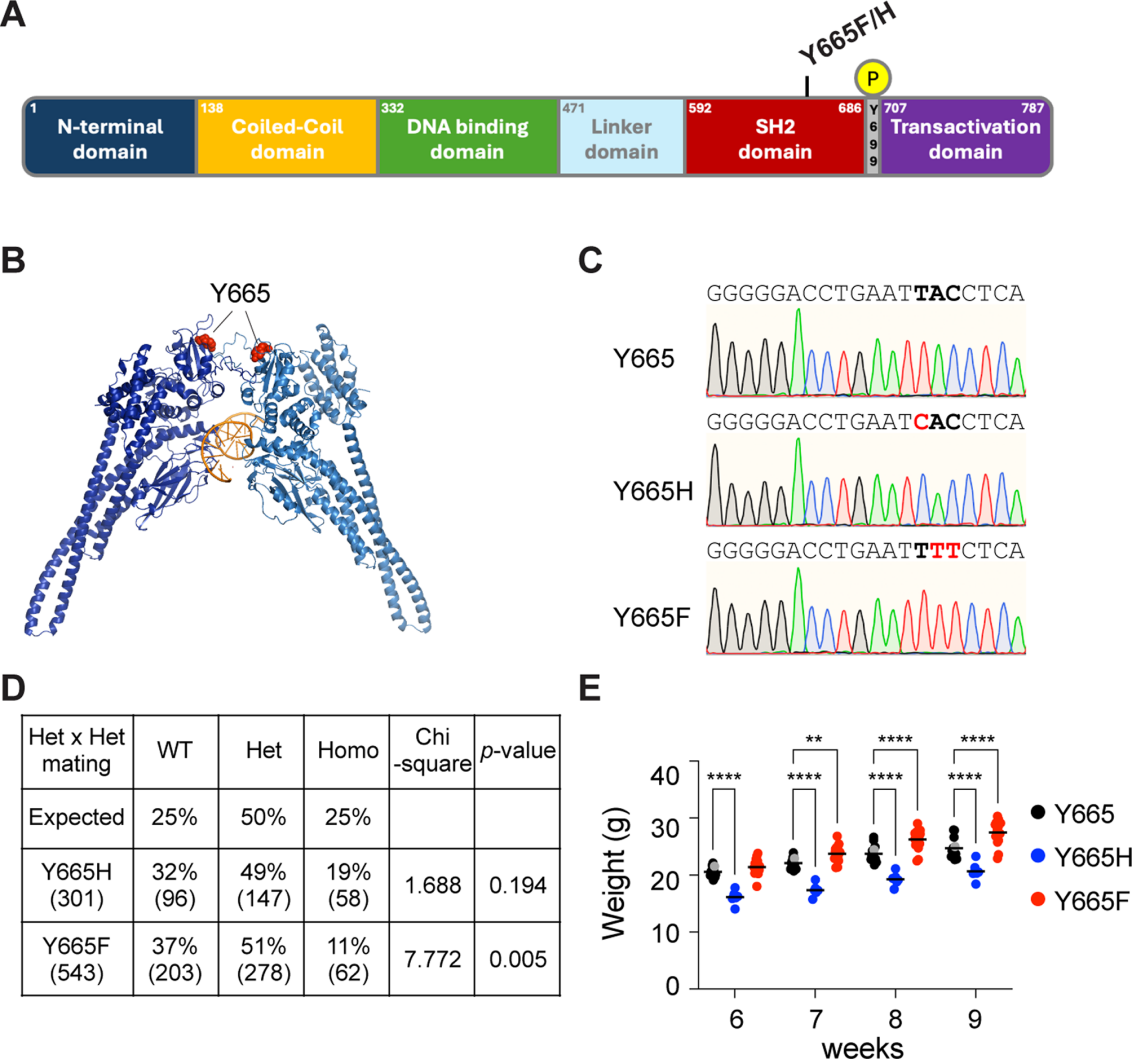
STAT5B mutations at Y665 in the mouse genome. **A** Schematic illustration of the STAT5B protein domains. Amino acid locations of the variants are highlighted. **B** Three-dimensional model of STAT5B protein structure presenting the location of the Y665 residue mutated in the mouse genome. **C** Sanger sequencing chromatograms showing Y665 (wild-type, WT) and the introduction of SNPs resulting in the two missense mutations, *Stat5b*^Y665*F*^ (Y665F) and *Stat5b*^Y665H^ (Y665H) mutants. The red shade indicates the altered codons converting Y665 to Y665F and Y665H, respectively. **D** Number and percentage of offspring from intercrossed heterozygote parents (Y665H, *n* = 301; Y665F, *n* = 543). The statistical relationship between the percentage and the expected Mendelian ratios was assessed using a Chi-square test. **E** Dot plot presenting the weight of individual mice in each group from 6, 7, 8 and 9 weeks of age. Wild-type mice are from litters weaned from heterozygous Stat5b^Y665H^ (gray) and Stat5b^Y665F^ (black) dams. Results are presented as the means ± SEM of independent biological replicates (Y665, *n* = 13; Y665H, *n* = 6; Y665F, *n* = 17). A two-way ANOVA followed by Tukey’s multiple comparisons test was used to evaluate the statistical significance of differences between groups. **p < 0.001, ****p < 0.0001

Here, we use experimental mouse genetics to investigate the biological impact of two human STAT5B mutations on genetic programs controlling mammary gland development during pregnancy and lactation. Two distinct SNPs result in the replacement of tyrosine 665 (Y665) within the SH2 domain with either phenylalanine (Y665F) or histidine (Y665H) (Fig. 1A, B). STAT5B^Y665^ is distinct from STAT5B^Y699^, the key tyrosine required for STAT5B dimerization and nuclear translocation [40]. Y665F replaces the sidechain with a more hydrophobic form of essentially the same type, possibly stabilizing the fold even more, thereby predicting a gain-of-function (GOF). In contrast, Y665H replaces the 6-membered ring with a 5-membered imidazole ring that is positively charged at physiological pH, possibly destabilizing the fold of the monomer, conceivably causing loss-of-function (LOF). Intriguingly, both the Y665F and Y665H mutations result in vastly prolonged activation of STAT5B upon cytokine stimulation [41], suggesting the possibility that they either hyper activate STAT5 target genes or promote aberrant transcription programs normally silent in cells carrying wild type alleles.

### Establishing Mice Expressing STAT5B^Y665F^ and STAT5B^Y665H^ Mutants

To understand the physiological impact of the STAT5B^Y665F^ and STAT5B^Y665H^ mutations on hormone-controlled genetic programs, we re-engineered the endogenous *Stat5b* locus using CRISPR/Cas9 and deaminase base editing and generated mice expressing the two STAT5B isoforms (Fig. 1C). Exome sequencing validated targeting precision and the absence of off-target mutations, including any within the closely related *Stat5a* gene. Lines of mutant mice were established and bred to homozygosity. Only 11% of the pups weaned from *Stat5b*^*Y665F*^ heterozygous crossings were homozygous for the mutation suggesting distinct embryonic or perinatal lethality, while homozygous and heterozygous mice derived from the *Stat5b*^*Y665H*^ heterozygous crossings showed close to expected Mendelian ratios (Fig. 1D).

In vivo, both mutant mice showed phenotypes demarcating them from wild-type mice. At the age of six weeks, the STAT5B^Y665H^ mice demonstrated significantly lower body weights as compared to littermate wild-type mice with the trend persisting through age nine weeks. STAT5B^Y665F^ mice showed a significantly higher body weights at age seven weeks and, again, the trend continued through age nine weeks as compared to littermate wild-type mice (Fig. 1E). STAT5B plays a crucial role in growth hormone signaling [6–9, 42], and our results indicate that the STAT5B^Y665F^ and STAT5B^Y665H^ missense mutations show the anticipated impact of inactivating as compared to activating *Stat5b* mutations, as predicted from the literature.

STAT5B is an essential transcription factor that primarily promotes immune cell survival and inhibits apoptosis, playing a key role in regulating immune cell development and function [43]. To assess STAT5 activity in mutant mice, we examined STAT5B protein levels and apoptosis activity in splenic T cells isolated from Y665F, Y665H, and WT (Y665) mice, following stimulation with interleukin (IL)-2 and IL-7 (Supplementary Fig. 1). Phospho-STAT5B (pSTAT5B), the activated form of STAT5B, was significantly higher in Y665F compared to WT, while phosphorylation was minimal in the Y665H mutant (Supplementary Fig. 1A). FACS analysis revealed decreased apoptosis in both CD4 + and CD8 + T cells of Y665F mice (Supplementary Fig. 1B), suggesting a survival advantage conferred by activated STAT5B in these cell types.

### STAT5B^Y665H^ Impedes Genetic Programs Controlling Mammary Development During Pregnancy

**Fig. 2:**
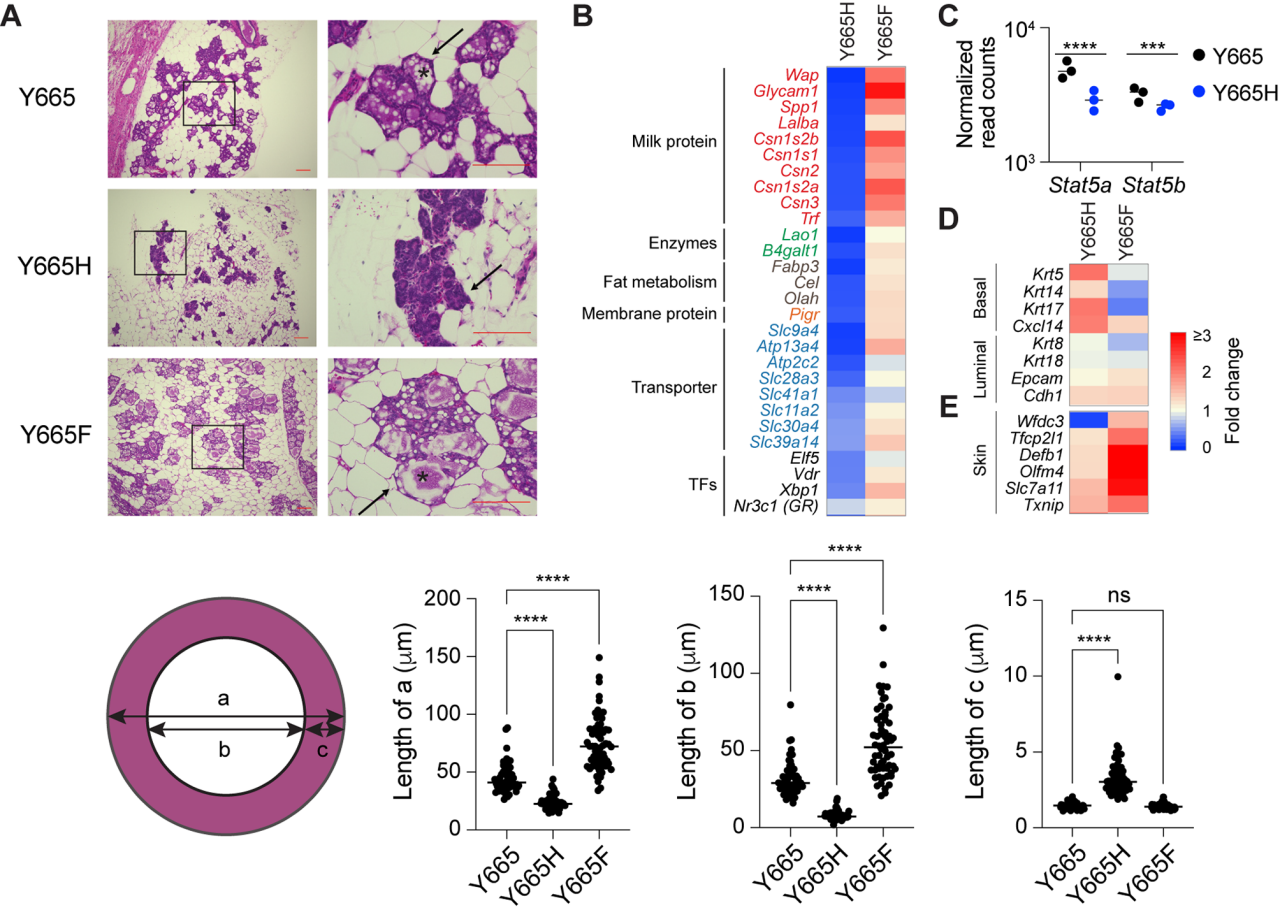
Impaired alveolar development in STAT5B^Y665^ mutant mice at day 18.5 of pregnancy. **A** Sections of mammary tissue stained with hematoxylin and eosin at day 18.5 of pregnancy with high magnification (x400, Scale bars, 100 μm). The arrow and Asterix indicates alveoli and lumen. Y665, *n* = 3; Y665H, *n* = 3; Y665F, *n* = 4. 50–100 of the alveolar architecture on each slide was quantitatively analyzed using the ImageJ software, with the results visualized through dot plots. (a) milk-secreting alveoli; (b) alveolar lumen; (c) alveolar epithelial cells. **B**, **D** and **E** Heatmaps showing fold activity of significantly regulated genes of milk proteins in red, enzymes in green, fat metabolism in gray, membrane transporters in blue, transcription factors in black (B), cell marker genes (D) and skin-related genes (E) in STAT5B^Y665H^ and STAT5B^Y665F^ mutants compared to WT (RNA-seq; Y665, *n* = 3; Y665H, *n* = 3; Y665F, *n* = 4). **C** Dot plots with the normalized read counts from RNA-seq to mRNA levels of *Stat5* genes controlled by autoregulatory enhancer. Results are shown as the means ± SEM of independent biological replicates (Y665, *n* = 3; Y665H, *n* = 3). The Benjamini-Hochberg adjusted *p*-value was used for significance. ***p < 0.0001, ****p < 0.0001

Although both the STAT5B^Y665F^ and STAT5B^Y665H^ mutation had been identified in human leukemic patients and showed activating features in tissue culture cells [41], they displayed divergent biological activity when introduced into the mouse genome. While homozygous STAT5B^Y665H^ females were unable to nurse their litters, STAT5B^Y665F^ dams successfully raised their pups, suggesting that the STAT5B^Y665H^ mutation exerts a negative impact on STAT5B function in mammary tissue during pregnancy. To understand possible molecular defects imposed by the STAT5B^Y665H^ mutation, we conducted histological and transcriptional analyses at day 18.5 of pregnancy (p18.5), one day prior to delivery (Fig. 2). Histological analyses revealed an undifferentiated mammary alveolar compartment and the absence of extended lumina and milk secretion (Fig. 2A). In contrast, mammary alveoli from wild type mice were characterized by open lumina filled with milk fat globules. We measured the alveolar and luminal diameters, and a significant increase was seen in STAT5B^Y665F^ mice (Fig. 2A). In contrast, both alveolar and luminal diameters were significantly decreased in STAT5B^Y665H^ mice, confirming the visual histological inspection. While the thickness of alveolar cells from wild type and Stat5B^Y665F^ mice were equivalent, it was significantly greater in STAT5B^Y665H^ mice supporting that these cells had failed to differentiate and secrete milk into the alveolar lumen, which would result in an alveolar expansion. Overall, these findings suggest that the STAT5B^Y665H^ mutation lacks biological activity, incapable of implementing functional differentiation of mammary alveoli and that the presence of STAT5A fails to compensate for the absence of a functional STAT5B, at least during the first pregnancy. Notably, mammary tissue from STAT5B^Y665H^ mice is distinctly different from mice lacking both STAT5A and STAT5B isoforms, which developed mammary ducts but failed to form alveoli and lacked true lobulo-alveolar units [3, 5]. This suggests that either the wild type STAT5A or residual activity of STAT5B^Y665H^ is sufficient for the establishment of an undifferentiated epithelium. In contrast to STAT5B^Y665H^, mammary tissue from STAT5B^Y665F^ mice had a normal histological appearance at day 18.5 of pregnancy (Fig. 2A), in line with mutant mice being able to nurse and raise their litters. If anything, STAT5B^Y665F^ mice displayed a more advanced differentiated alveolar compartment.

Genes encoding milk proteins and other components controlling milk production and secretion during lactation, such as membrane transporters and enzymes establishing lipid homeostasis, are activated up to several-thousand-fold during pregnancy, a process largely controlled by the combined action of JAK2 [19] and STAT5A/B [14]. To determine to what extent expression of these genes was impacted by the STAT5B^Y665H^ and STAT5B^Y665F^ variants, we conducted RNA-seq from mammary tissue collected at day 18.5 of pregnancy, just prior to parturition. Expression of approximately 660 genes related to organic acid and metal ion transport, nucleoside phosphate metabolic processes, and fatty acid biosynthesis was reduced by at least 50% in STAT5B^Y665H^ mice (Supplementary Table 3). To gauge the direct impact of STAT5B^Y665H^ on mammary epithelial differentiation, we focused on three functional gene classes, milk protein genes, membrane transporters contributing to milk secretion and regulatory proteins (receptors, transcription factors, enzymes). Expression of milk protein genes, that comprise more than 95% of all mRNAs, was greatly reduced, ranging from a more than a 90% decline for genes, such as *Wap* and *Glycam1*, to a more modest 50% for membrane transporters (Fig. 2B; Supplementary Table 3). STAT5A/B not only controls the expression of milk protein, but also activates transcription factor genes, such as *Elf5*, *Vdr*, *Xbp1* and *Nr3c1* that are known to participate in the activation of mammary genetic programs. Expression of these genes was reduced by 50–60% (Fig. 2B; Supplementary Table 1). Autoregulation of the *Stat5a/b* locus is a notable feature that facilitates its critical role in the activation of mammary genetic programs [44] and reduced *Stat5a/b* expression was observed in STAT5B^Y665H^ mice (Fig. 2C) suggesting that this mutant had an impaired biological activity. Lastly, expression of members of the solute carrier (SLC) protein family that are increased during lactation to transfer ingredients with high nutritional interest into milk [45, 46] is greatly diminished, possibly explaining the absence of open mammary alveolar lumina and milk (Fig. 2B). Findings also suggest that the impaired transcriptional activity of STAT5B^Y665H^ cannot be sufficiently compensated for by the presence of a wild type STAT5A.

### STAT5B^Y665F^ Augments Mammary Differentiation During Pregnancy

In contrast to STAT5B^Y665H^, STAT5B^Y665F^ mice exhibited accelerated mammary differentiation at day 18.5 of pregnancy as evidenced by histological appearance and increased alveolar and luminal size (Fig. 2A). This was further supported by an elevated expression of some milk protein genes, such as *Csn2* (Fig. 2B; Supplementary Table 4). However, this increase was less than 2-fold, in sharp contrast to the 95% reduction seen for some genes in STAT5B^Y665H^ mice. While we found that basal cell markers are elevated in STAT5B^Y665H^ mice, the expression levels of luminal epithelial cell markers are similar in both mutants compared to wild-type (WT) mice (Fig. 2D). This suggests that the observed alterations in gene expression are likely due to significant differences in mammary differentiation, rather than significant differences in the luminal epithelial cell population. Unexpectedly, STAT5B^Y665F^ activated a set of approximately 140 genes that are normally not under the control by pregnancy hormones through JAK-STAT. These genes are involved in innate and adaptive immune responses, T cell activation, and fatty acid metabolic processes. Among those, skin-related genes, including *defensin beta 1* (*Defb1*), are activated more than 2 to 20-fold in STAT5B^Y665F^ mice, but to a lesser extent in STAT5B^Y665H^ mice (Fig. 2E, Supplementary Fig. 2, Supplementary Table 4, Source file). These findings suggest that the STAT5B^Y665F^ mutation has a modest positive impact on *bona fide* mammary-specific genes but aberrantly activates gene classes that are not under pregnancy control, including genes key for skin homeostasis.

### STAT5B^Y665F^ and STAT5B^Y665H^ Differentially Impact Mammary Enhancers

**Fig. 3:**
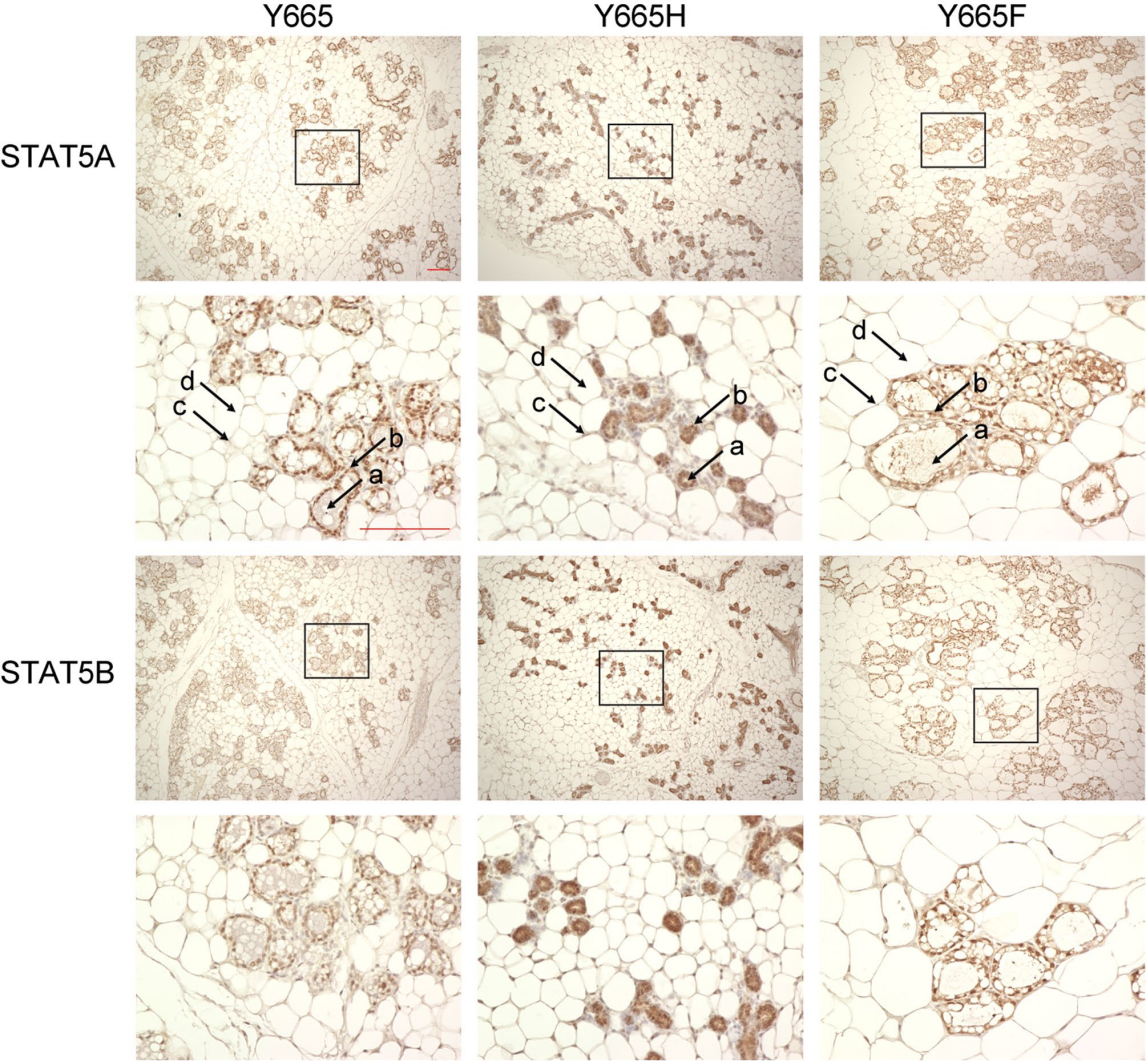
Nuclear localization of STAT5A and STAT5B in STAT5B^Y665^ mutant mice. Staining of STAT5A and STAT5B proteins at day 18.5 of the first pregnancy with high magnification (x10 and x400, Scale bars, 100 μm). The arrow indicates alveolar lumen (**a**), alveolar epithelial cells (**b**), fibroblasts (**c**) and (**d**) fat

Upon their activation in the cytoplasm during pregnancy, STAT5A/B translocate to the nucleus where they establish mammary enhancers and super-enhancers [14–17] and thereby promote gene transcription. Mechanistically, the reduced activity of the STAT5B^Y665H^ isoform could be explained by a diminished nuclear translocation or a failure to build an enhancer landscape and activate genetic programs. Conversely, the STAT5B^Y665F^ isoform might lead to augmented nuclear translocation and enhancer occupation. We addressed these possibilities through immunohistochemical and ChIP-seq analyses. Immunohistochemistry was performed on mammary tissue collected at day 18.5 of pregnancy (Fig. 3). Although nuclear staining was observed in wild-type tissue and both mutants, it was distinctly nuclear in the wild type and STAT5B^Y665F^ mice. In contrast, STAT5 protein was also present in the cytoplasm of STAT5B^Y665H^ tissue, suggesting that the missense mutation affects the nuclear translocation of STAT5B.

**Fig. 4:**
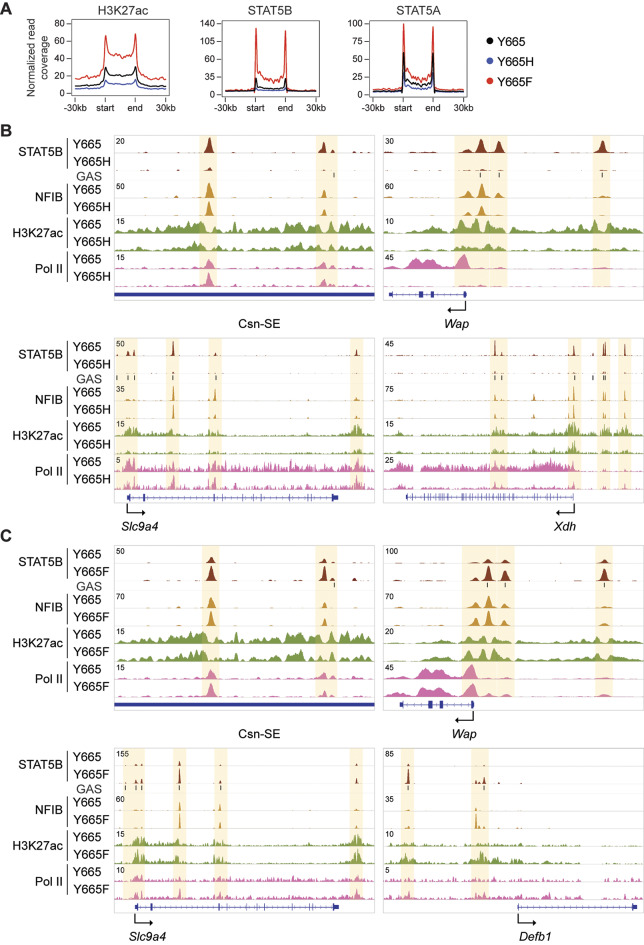
Genomic features of the genes regulated by STAT5B^Y665^ mutations. **A** Coverage plots displaying the patterns of H3K27ac marks and STAT5B and STAT5A binding on 440 mammary-specific super-enhancers at day 18.5 of pregnancy. Black, Y665; blue, Y665H; red, Y665F. **B** Binding of STAT5B and NFIB, H3K27ac, and Pol II at gene loci of milk protein genes controlled by mammary-specific super-enhancers, transporter genes, and skin genes in mammary tissue of wild type (Y665) and *Stat5b*^*Y665H*^ (Y665H) mice at day 18.5 of pregnancy. **C** STAT5B and NFIB binding, H3K27ac and Pol II loading at gene loci of milk protein genes controlled by mammary-specific super-enhancers, transporter gene and skin gene in mammary tissue of Y665 and *Stat5b*^*Y665F*^ (Y665F) mice. Y665, *n* = 2; Y665H, *n* = 2; Y665F, *n* = 2

Next, we asked if the STAT5B^Y665H^ mutant displays compromised binding to the respective DNA binding sites (GAS motifs, TTCnnnGAA), thereby failing to activate mammary enhancers. We also investigated whether the elevated expression of target genes in STAT5B^Y665F^ mice was due to enriched binding to enhancers. Towards this end, we conducted ChIP-seq experiments and interrogated the capacity of STAT5B^Y665H^ and STAT5B^Y665F^ to recognize GAS motifs within enhancers linked to mammary-specific and more widely expressed STAT5A/B target genes (Fig. 4). While STAT5B binding to mammary-specific enhancers, such as those driving the *Casein* locus [17] or the *Wap* gene [15] was detected in tissue collected from wild type mice at day 18.5 of pregnancy, little or no binding was observed in tissue collected from STAT5B^Y665H^ mice. The absence of STAT5B binding paralleled reduced H3K27ac coverage and Pol II loading (Fig. 4A), supporting their reduced expression levels. As outlined above, the remaining expression of milk protein genes observed in STAT5B^Y665H^ mice might be the result of other transcription factors, such as NFIB, known to bind to mammary enhancers [15, 17, 47], a concept we tested using ChIP-seq experiments. Notably, NFIB binding to the *Casein*, *Wap*, *Slc9a4* and *Xdh* enhancers was not significantly reduced in STAT5B^Y665H^ mice (Fig. 4B), establishing NFIB as a transcription factor that can function in the presence of a transcriptionally impaired STAT5B.

Next, we examined whether the gene activation observed in STAT5B^Y665F^ mice could be explained by an enhanced binding of the mutant STAT5B to mammary enhancers and super-enhancers. Increased STAT5B binding to known mammary enhancers was observed in STAT5B^Y665F^ mice (Fig. 4C). Importantly, strong STAT5B binding was observed at candidate enhancers of the *de novo* induced genes, such as *Defb1* (Fig. 4C) whose expression was induced 17-fold. Concordantly, NFIB binding was enhanced suggesting either an indirect recruitment through STAT5B or binding to NFIB motifs in a more optimal accessible chromatin.

### STAT5B^Y665F^ Promotes a Temporal Shift in Mammary Differentiation during Pregnancy

**Fig. 5:**
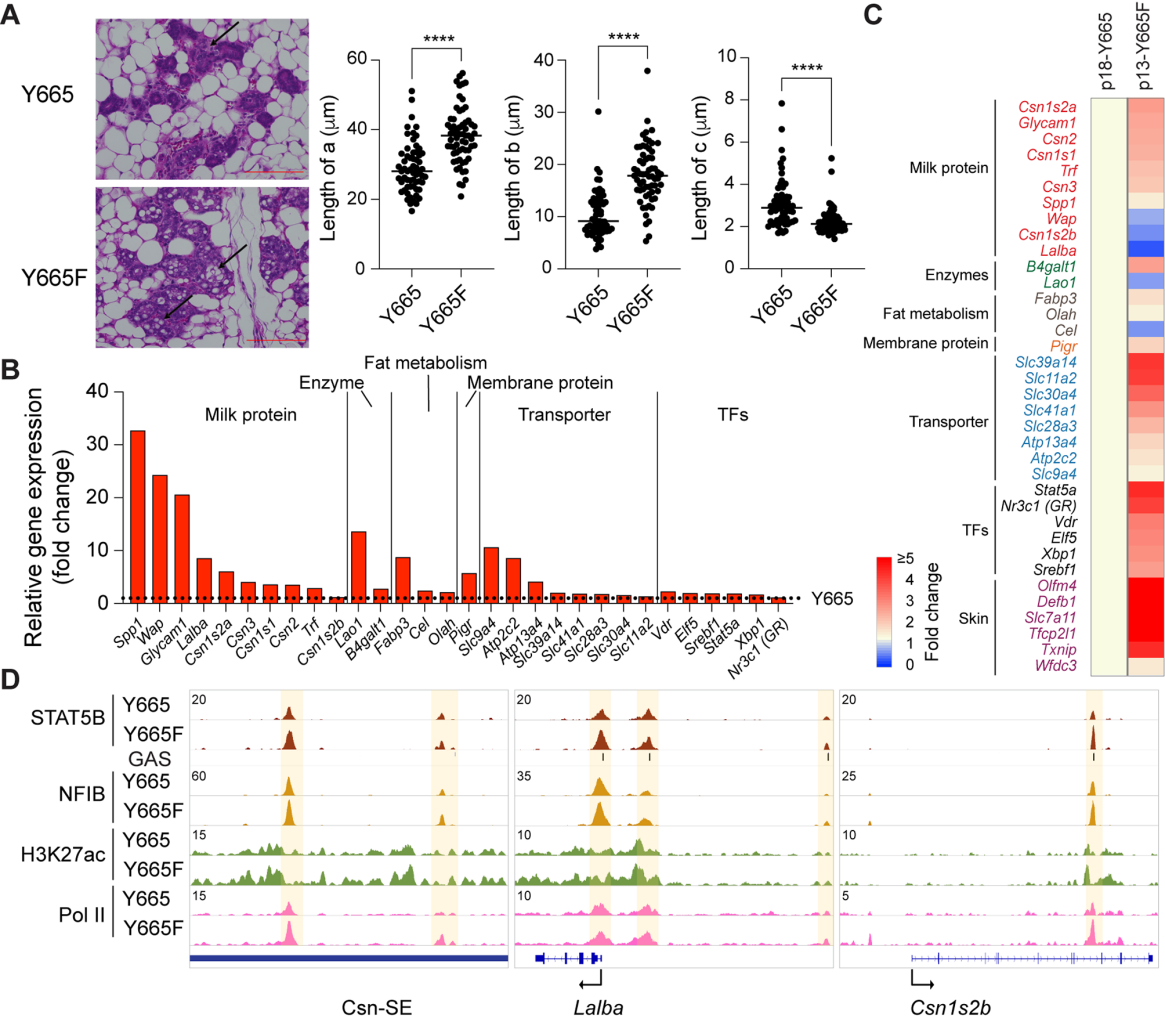
Alveolar development of STAT5B^Y665F^ mutants at mid-pregnancy. **A** Sections of mammary tissue stained with hematoxylin and eosin at day 13.5 of pregnancy with high magnification (x400, Scale bars, 100 μm). The arrow indicates alveoli. Y665, *n* = 3; Y665F, *n* = 4. The alveolar architecture was quantitatively analyzed using the ImageJ software, with the results visualized through dot plots. (a) milk-secreting alveoli; (b) alveolar lumen; (c) alveolar epithelial cells. **B** Bar graphs showing fold activity of genes of milk proteins, transporters and transcription factors in STAT5B^Y665F^ (Y665F) mice compared to wild type (Y665) (RAN-seq; Y665, *n* = 3; Y665F, *n* = 4). **C** Heatmaps presenting fold activity of milk protein genes in red, transporter genes in blue, transcription factors in black (left) and skin genes (right) in STAT5B^Y665F^ mice at day 13.5 of pregnancy (presented as p13) compared to WT at day 18.5 of pregnancy (presented as p18) (RAN-seq; Y665 at p18, *n* = 3; Y665F at p13, *n* = 4). **D** Binding of STAT5B and NFIB, H3K27ac, and Pol II at gene loci of milk protein genes controlled by mammary-specific super-enhancers in mammary tissue of Y665 and Y665F mice at day 13.5 of pregnancy. Y665, *n* = 2; Y665F, *n* = 2

Genes encoding milk proteins and other regulatory proteins display distinct temporal activation patterns in mammary tissue with a sharp increase of transcription frequently observed in the latter part of pregnancy [14, 18]. Since STAT5B^Y665F^ is a *bona fide* activating mutation, we asked if it would lead to a temporal shift of the differentiation program during pregnancy. For this, we investigated the histological appearance and transcription programs of mammary tissue at day 13.5 of pregnancy (p13.5), a stage when key mammary genes have not been activated due to suboptimal STAT5A/B signaling. Histologically, STAT5B^Y665F^ tissue displayed an advanced degree of development and differentiation as indicated by an accumulation of lipid droplets and open lumina (Fig. 5A). Alveolar and luminal diameter of mutant tissue were larger than those measured in wild type tissue (Fig. 5A). The precocious expansion of mutant alveoli also resulted in stretched and thinner secretory epithelium.

Transcriptome analysis revealed a sharp increase of milk protein gene transcripts, such as *Spp1* and *Wap*, the mammary-centric transcription factor *Elf5* and membrane transporters required for secretion of milk (Fig. 5B; Supplementary Table 5). Expression of some of these genes approached levels seen at day 18.5 of pregnancy (p18.5) in wild type (Fig. 5C), confirming precocious differentiation. ChIP-seq experiments confirmed increased STAT5B binding to key mammary enhancers (Fig. 5D).

Having observed that mammary alveolar differentiation is shifted to mid pregnancy in STAT5B^Y665F^ mice, we asked whether the activated STAT5B also induces mammary-specific genes in non-parous mice (Supplementary Fig. 3). First, we prepared whole mounts and investigated the structural features of mammary ducts in 2–3 months-old wild type and mutant mice. Ductal elongation and branching were similar in all genotypes, filling the entire fat pad (Supplementary Fig. 3A). However, ducts appeared to be thinner in all STAT5B^Y665F^ mice analyzed (Supplementary Fig. 3B). Expression of milk proteins and other differentiation associated genes is not confined to alveoli but has also been shown in ductal epithelium [48]. To gauge the differentiation status of the ductal epithelium in non-parous mice we measured the expression of a set of milk protein genes (Supplementary Fig. 3C). Expression of key milk protein genes increased more than 30-fold in STAT5B^Y665F^ mice, while their expression decreased in STAT5B^Y665H^ mice. Expression of *Cish*, a STAT5-controlled gene expressed across many cell types, was unaltered.

### Physiological Adaptation of STAT5B^Y665H^ Mice After Successive Pregnancies

**Fig. 6:**
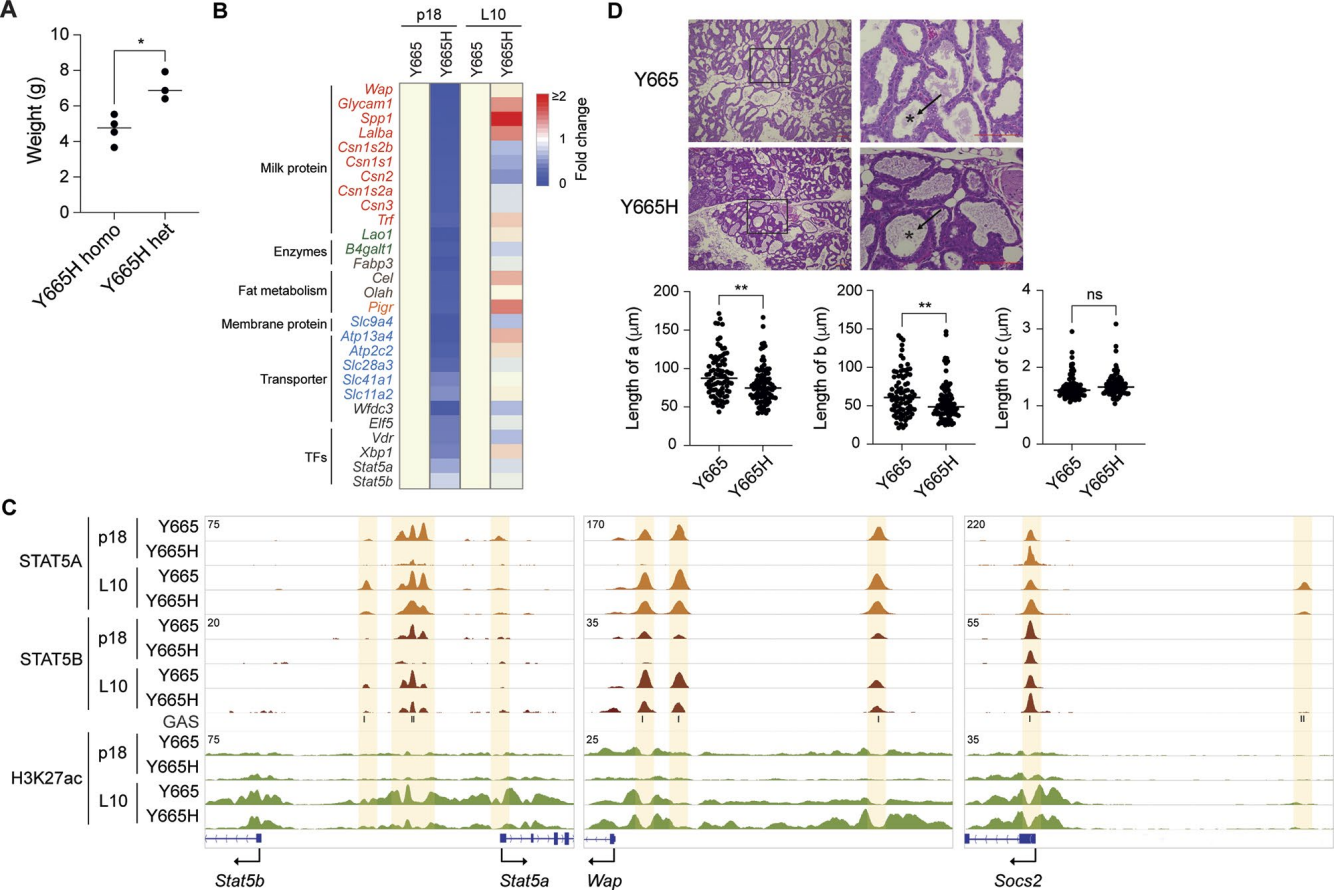
Partial activation of mammary function and lactation in STAT5B^Y665H^ mice after the second pregnancy. **A** Body weight of pups at day ten of lactation (L10) after the second pregnancy of homozygous *Stat5b*^*Y665H*^ mice and after the first pregnancy of heterozygous *Stat5b*^*Y665H*^ mice. The weight of pups from 3–4 females was measured and normalized by the number of pups. A *t*-test was utilized to evaluate the statistical significance between pups from homozygous females at second pregnancy and heterozygous females at first pregnancy. Homozygous *Stat5b*^Y665H^ mice, *n* = 4 cages; heterozygous *Stat5b*^*Y665H*^ mice, *n* = 3 cages. *p < 0.05. **B** Expression of representative mammary genes was measured in mammary tissue of Y665 (WT) mice collected at day 10 of lactation (L10), and of STAT5B^Y665H^ mice at L10 after the second pregnancy by RNA-seq (Y665, *n* = 3; Y665H, *n* = 4). The p18 data (Fig. 2) is presented alongside the L10 data to highlight the relative reduction in gene expression between p18 and L10 in Y665H mice. **C** Binding of STAT5A and STAT5B, H3K27ac, and Pol II at *Stat5* and *Wap* super-enhancer loci controlled by prolactin in mammary tissue of Y665 and Y665H mice at day 10 of lactation. The *Socs2* locus was presented as a ChIP-seq control. Y665, *n* = 2; Y665H, *n* = 2. **D** Sections of mammary tissue stained with hematoxylin and eosin at day 10 of lactation after the second pregnancy with high magnification (x400, Scale bars, 100 μm). The arrow and Asterix indicates alveoli and lumen. Y665, *n* = 1; Y665H, *n* = 3. The alveolar architecture was quantitatively analyzed using the ImageJ software, with the results visualized through dot plots. (a) milk-secreting alveoli; (b) alveolar lumen; (c) alveolar epithelial cells

Although prolactin exposure during the 18.5 days of the first pregnancy was not sufficient to execute functional mammary development in STAT5B^Y665H^ mice, it was unclear whether continued hormonal stimulation would overcome the developmental block. To test this, we mated homozygous mutant female mice immediately after their first pregnancy, thereby enabling a continued prolactin stimulus on mammary epithelium. Mutant dams were able to support litters after their second pregnancy, although the pups grew slower (Fig. 6A), possibly the result of a reduced milk production. To gauge the differentiation status of mammary tissue, and thereby indirectly milk protein production, we measured mRNA levels of distinct milk protein mRNAs at the end of the first pregnancy (p18.5) and at day 10 of lactation (L10) after the second pregnancy using RNA-seq (Fig. 6B). While the expression of key milk protein genes was reduced by up to 97% at the end of the first pregnancy of STAT5B^Y665H^ dams as compared to control mice, expression levels were approximately 50% of those observed in control mice after the second pregnancy (Fig. 6B; Supplementary Table 6). Notably, the expression of some genes, such as *Glycam1*, *Spp1* and *Lalba*, was higher in mutant tissue.

To understand the impact of the second pregnancy and lactation on the establishment of enhancer structures, we conducted ChIP-seq on mammary tissue collected at L10. Binding of STAT5A and STAT5B to mammary enhancers and H3K27ac were equivalent in mutant and control mammary tissue (Fig. 6C). Histological analysis corroborated these findings, revealing the presence of functional mammary tissue with differentiated alveoli containing milk-filled lumina (Fig. 6D). However, alveolar and luminal size in mutant tissue did not approach those in control tissue (Fig. 6D).

### Fatty Acid Metabolism Genes are Controlled by STAT5B

**Fig. 7:**
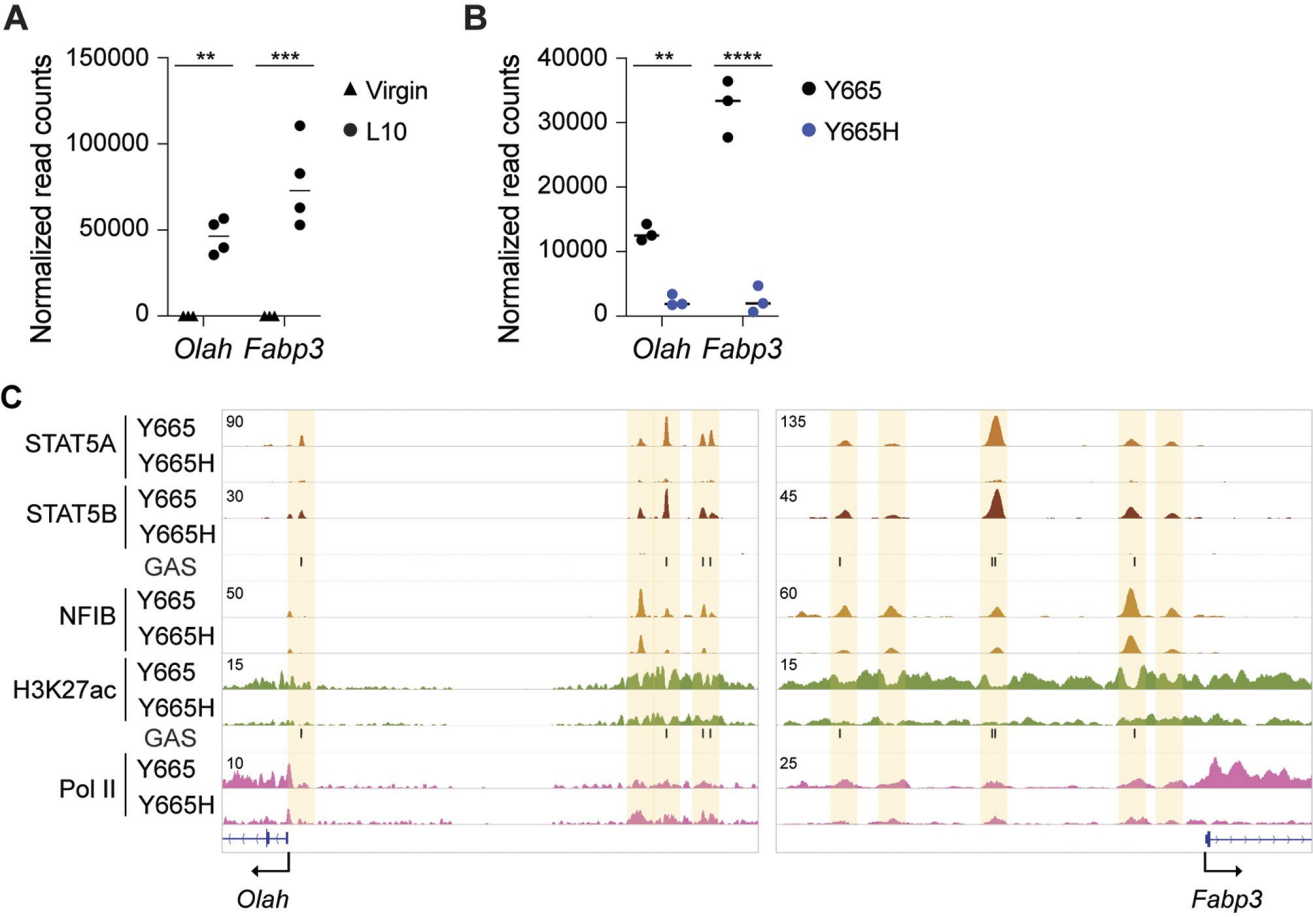
Regulation of key fat metabolic genes. **A-B** Dot plots with the normalized read counts from RNA-seq to mRNA levels of *Olah* and *Fabp3* genes at virgin and L10 of wild type mice (A) and at p18 of wild type and Y665 mutant mice (B). Results are shown as the means ± SEM of independent biological replicates (WT at virgin, *n* = 3; WT at L10, *n* = 4; Y665 at p18, *n* = 3; Y665H at p18, *n* = 3). *p*-values are from 2-way ANOVA followed by Sidak’s multiple comparisons test between WT and mutants. **p < 0.001, ****p < 0.0001. **C** Binding of SAT5A and STAT5B, H3K27ac, and Pol II at *Olah* and *Fabp3* gene loci controlled by prolactin in mammary tissue of Y665 and Y665H mice at day 10 of lactation. Y665, *n* = 2; Y665H, *n* = 2

A recent publication linked high expression of oleoyl-ACP hydrolase (*Olah*) to life-threatening respiratory illnesses, particularly COVID-19 [49]. In severely ill COVID-19 patients, elevated levels of OLAH were observed. Fatty acid binding protein 3 (FABP3) is another enzyme critical for fatty acid metabolism in lactating mammary epithelium [50, 51]. However, regulation of these genes had not been explored. Given their high expression in mammary tissue, particularly during lactation, we explored their regulation. Expression of the *Olah* and *Fabp3* genes increased approximately 6,500 or 4,000-fold during pregnancy and lactation, respectively (Fig. 7A), indicating regulation by pregnancy hormones via STAT5A/B. To test this hypothesis, *Olah* and *Fabp3* expression was examined in STAT5B^Y665H^ mutant tissue by day 18.5 of pregnancy. The observed reduction in their mRNA levels of over 80% mirrors that of the casein genes, known STAT5 target genes (Fig. 7B). Robust STAT5A/B binding was demonstrated at *Olah* promoter sequences and a four-partite upstream region (Fig. 7C), resembling the *Casein* locus super-enhancer [17]. Consistent with the reduced expression observed in STAT5B^Y665H^ mice, STAT5A/B binding to the candidate regulatory elements was largely diminished. By day 10 of lactation following the second pregnancy, both *Olah* expression and STAT5 binding were restored to wild-type levels (Fig. 6B). STAT5B^Y665F^ precociously induced *Olah* expression at day 13 of pregnancy (Fig. 5C), further supporting the contribution of STAT5A/B. This pattern of *Olah* regulation during pregnancy and lactation mirrors that of milk protein genes, which have STAT5-dependent enhancers [15, 44].

### STAT5A/B Dimerization Is Impaired in STAT5B^Y665H^ Mice

**Fig. 8:**
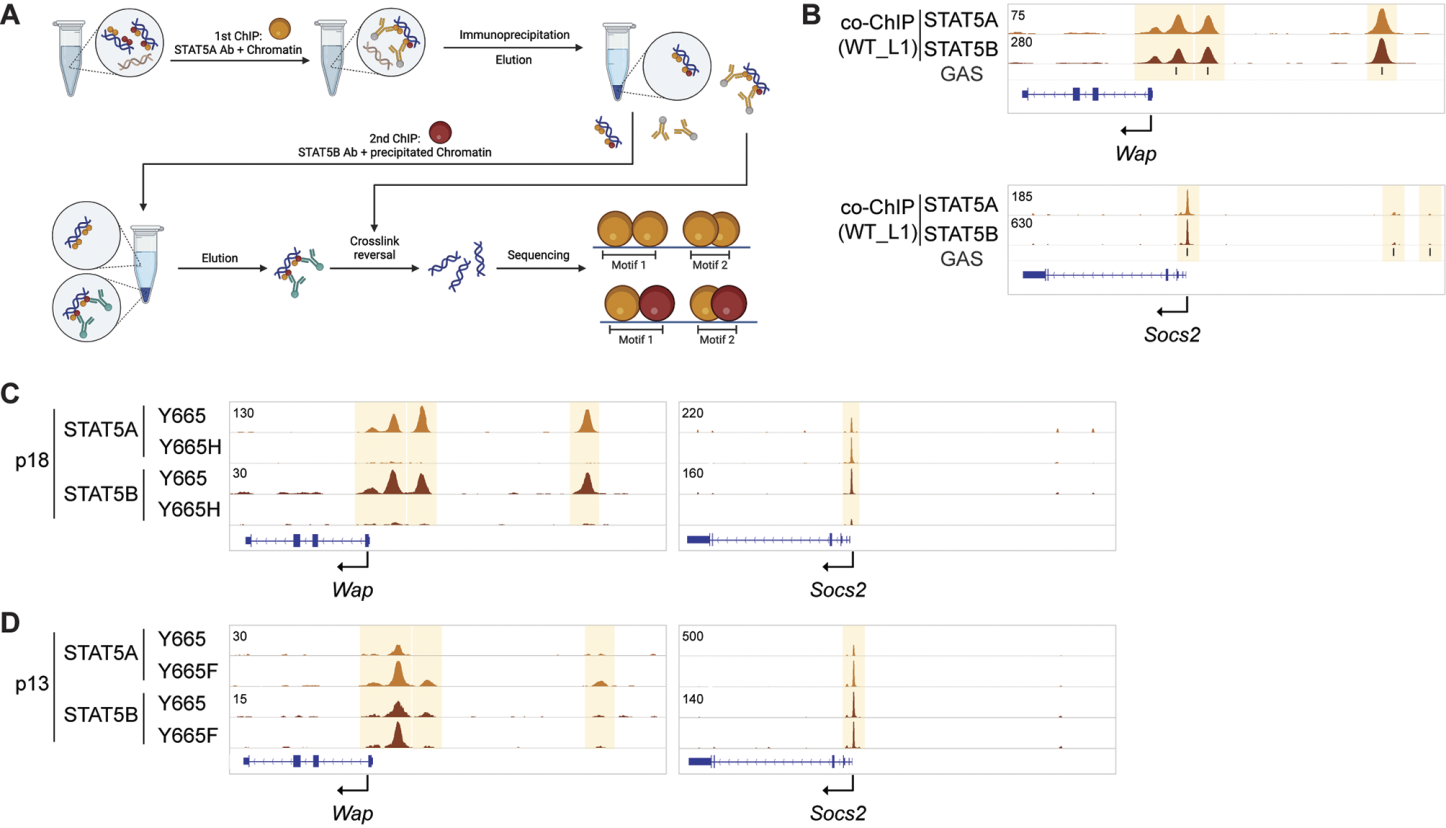
Dimerization of STAT5A and STAT5B protein. **A** Strategy of co-ChIP-seq. STAT5A bound chromatin from mammary tissue of WT mice at day 1 of lactation (L1) was precipitated using STAT5A antibody and eluted from Ig beads. Secondly, STAT5B bound chromatin was precipitated using STAT5B and eluted from the beads. STAT5A motifs from 1st ChIP and STAT5B motifs from 2nd ChIP were identified by sequencing. **B** Co-ChIP profile at the representative milk protein *Wap* gene and common STAT target *Socs2* gene. **C** STAT5A and STAT5B binding at gene loci of milk protein *Wap* gene and common STAT target *Socs2* gene in the mammary gland of Y665 (WT) and Y665H mice at day 18.5 of pregnancy (p18). **D** STAT5A and STAT5B binding in the mammary gland of Y665 (WT) and Y665F mice at day 13.5 of pregnancy (p13)

STAT proteins are known to form homodimers and heterodimers through bivalent SH2-pTyr interactions [40, 52–54], where the C-terminal phosphotyrosine, located in the transactivating domain of each STAT5 monomer, binds to the SH2 domain of the other. This interaction enables STAT5 dimers to recognize GAS motifs and drive diverse transcriptional responses. We had originally hypothesized that STAT5A would compensate, at least in part, for the absence of a functional STAT5B. However, the greatly impaired expression of milk protein genes in STAT5B^Y665H^ mice suggested the inability of STAT5A to compensate for the defective STAT5B. The possibility arose that STAT5A cannot activate mammary target genes by itself or, alternatively, STAT5A binding to its target sites in enhancers is dependent on a functional STAT5B. To address these options, we first confirmed the presence of STAT5A and STAT5B heterodimers in mammary tissue using ChIP-seq. For this we conducted a two-step ChIP-seq experiment. In the first step, chromatin was precipitated using STAT5A-specific antibodies, followed by elution. Secondly, the eluted chromatin, precipitated by the STAT5A protein, was then subjected to precipitation again using STAT5B-specific antibodies (Fig. 8A). This experiment demonstrated that STAT5A/B heterodimers occupy mammary-specific and common enhancers (Fig. 8B).

Next, we investigated if STAT5A binding was impacted by the STAT5B^Y665H^ mutant and conducted ChIP-seq experiments from mammary tissue harvested at day 18.5 of pregnancy (Fig. 8C). Notably, in addition to the failure of STAT5B^Y665H^ to bind to mammary enhancers, no STAT5A binding was detected in the *Wap* enhancers, suggesting that STAT5A/B heterodimers are the major complex recognizing these enhancers. In contrast, STAT5A binding to common STAT5-controlled enhancers in *Socs2* was not visibly affected (Fig. 8C). Based on these results it could be predicted that enhanced STAT5B^Y665F^ activity would recruit additional STAT5A to enhancers. To test this, we conducted STAT5A ChIP-seq in STAT5B^Y665F^ tissue at day 13.5 of pregnancy (Fig. 8D). Elevated STAT5A occupancy was detected at mammary enhancers in STAT5B^Y665F^ mice, as indicated at the *Wap* gene enhancers, as compared to control tissue.

## Discussion

The insertion of the two human mutations, STAT5B^Y665F^ and STAT5B^Y665H^, into the mouse genome permitted a robust comparative investigation and uncovered unexpectedly intricate properties of the two variants that had previously been identified in human T cell leukemias. Tyrosine 665 within the STAT5B SH2 domain is essential for successful mammary gland development, homeostasis and lactation. Our study exposed that the two mutations elicited distinctly opposite effects on transcription enhancers and super-enhancers resulting in altered genetic programs controlling mammary development and lactation. While the STAT5B^Y665F^ mutation enhanced STAT5B activity, it was greatly reduced by the STAT5B^Y665H^ mutation. The impact of the STAT5B^Y665H^ mutation varied markedly between the first and second pregnancy, emphasizing the importance of persistent stimulation by pregnancy hormones in overcoming partially inactivating mutations, possibly through upregulating the compensating STAT5A, and attaining functional mammary development and lactation.

Both mutations had initially been identified in human leukemic patients [21, 22], and tissue culture cell experiments [41] categorized them as activating ones. Unexpectedly, *Stat5b*^*Y665H*^ proved to be an inactivating mutation in our study, while *Stat5b*^*Y665F*^ showed enhanced activity as would have been expected from in vitro studies [41]. The apparently contradictory results obtained with the STAT5B^Y665H^ variant may be due to differences in experimental approaches, here the use of gene editing to knock in the mutation into the endogenous *Stat5b* locus while the tissue culture cell experiments were based on retroviral mediated overexpression. The functional impact of the two mutations in vivo also highlights the role of STAT5B in mediating sexually dimorphic growth, the activating mutation accelerating and the inactivating one retarding growth in males [42].

The full in vivo significance of the two mutations was uncovered by examining their impact on the physiological process of mammary development and lactation, processes controlled by maximal physiological prolactin stimulation during pregnancy. While STAT5B^Y665H^ did not support functional mammary development during pregnancy, STAT5B^Y665F^ induced precocious mammary development. Mechanistically, diminished STAT5B function resulted in failure to establish functional enhancer complexes throughout the mammary genome, thereby limiting transcriptional activity of genes required for milk production by more than 90%. Greatly reduced expression of genes encoding milk proteins, membrane transporters and enzymes controlling lipid homeostasis can explain the failure of lactation in mutant mice.

Importantly, while a single round of pregnancy failed to activate a lactational program in STAT5B^Y665H^ mice, successive pregnancies supported functional mammary development and lactation. This finding illustrates the power of functional adaptation in, at least partially, overriding programs devoid of normal levels of *Stat5a* [55] or the prolactin receptor (*Prlr*) [56]. Continued prolactin signaling through two pregnancies also restores a normal mammary enhancer landscape and transcription programs observed in the presence of a normal STAT5B. We suggest that several independent mechanisms foster the establishment of functional mammary development after two pregnancies. First and foremost, an adjunct mammary epithelial cell population formed during pregnancy has been linked to lactational differences observed in parous females and suggested to facilitate functional adaptation [56]. Parity-induced changes in mammary cell populations also include immune cells, including natural killer T cells [57], whose physiology could be impacted by the STAT5B^Y665H^ variant. DNA methylation studies further support that epithelial cells undergo substantial changes during the first pregnancy, thereby permitting them to respond more efficiently during subsequent pregnancies [58]. Combined, these findings might help explain differences in human lactational performance after the second pregnancy [59].

The STAT5B^Y665F^ variant led to a temporal shift in alveolar differentiation towards mid pregnancy. Significantly, even non-parous mice showed elevated levels of milk protein transcripts further supporting the activating nature of this mutation. Functionally, the advanced mammary development during pregnancy likely reflects precocious activation of key mammary enhancers and super-enhancers at early stages of pregnancy as well as during specific phases of the estrous cycle in virgin mice. Hyperactive variants, such as STAT5B^Y665F^, can access native chromatin that is otherwise inactive due to its methylation and acetylation status [60]. Transcriptional analyses showed that this missense mutation not only induced native mammary-centric genetic programs, but it also stimulated genes that are silent during normal development. This finding demonstrates that activating STAT5B variants can induce aberrant genetic programs with implications for mammary cancer and immune homeostasis [61].

STAT5A, originally called mammary gland factor (MGF) [10], is critical for functional differentiation of mammary epithelium during pregnancy and lactation [4]. Specific study of the contribution of STAT5B to this process has been hampered by the infertility of the corresponding knockout mice [42]. An important contribution of our study is to define STAT5B as a critical isoform in driving pregnancy-mediated mammary development and highlighting its important function as a heterodimer with STAT5A. A preferential heterodimerization of Stat5A/Stat5B may impose tissue-specific or developmentally mediated functions, as under in vitro condition in T cells this is not observed [62, 63]. Bivalent SH2-pTyr interactions are required for stable STAT5A/B heterodimerization and the introduction of STAT5A^Y694F^ and STAT5B^Y699F^ mutations impedes heterodimerization. Although both STAT5B^Y699F^ and STAT5B^Y665H^ are inactivating mutations, they have distinct impacts on mammary development and possibly also immune cells. While homozygous STAT5B^Y699F^ females support their offspring, STAT5B^Y665H^ homozygosity results in lactation failure. It can be hypothesized that the STAT5B^Y665H^ missense mutation has a broader defect, also preventing STAT5A binding to enhancers as shown in our study.

STAT protein SH2 domains support JAK2-mediated phosphorylation, dimerization, nuclear translocation and binding to regulatory elements in promoters and enhancers. In humans, missense mutations have been identified at approximately 30 amino acids within the STAT5B SH2 domain, falling into three categories: candidate gain-of-function (GOF), loss of function (LOF) and dominant negative. STAT5B SH2 mutations associated with the growth hormone insensitivity (GHI) syndrome include A630P, K632N, F646S and V669F result in postnatal growth failure and, in some cases, immune deficiency [8, 64, 65]. They are considered to disrupt the anti-parallel β-sheets and thereby the pocket for binding phosphate groups on respective receptors, resulting in abrogated STAT5 activation. The STAT5B^Y665H^ variant studied here would also be predicted to result in disruption of the anti-parallel β-sheets and abrogation of STAT5B function, observed experimentally here.

Women who experience suboptimal milk production with the first pregnancy can find that lactation improves following the second pregnancy [59]. Although the genetic basis for this phenomenon has not been widely investigated, mutations in the prolactin gene have been identified in women with puerperal lactogenesis [66]. These inactivating mutations would inevitably result in a suboptimal activation of STAT5A/B and subsequent difficulties in maintaining lactation. Additional individuals with prolactin gene mutations can be identified in general population databases such as AllofUs, providing the opportunity to investigate the role of genetic variations in lactational diversity. In mice, an activating mutation in the viral sensor 2’-5’- oligoadenylate synthetase 2 (*Oas2*) gene leads to lactational failure [67] in association with induction of immune pathway genes in mammary alveolar epithelium. Since *Oas2* is a STAT5A/B target [68, 69], it can be hypothesized that mutations in other genes under STAT5A/B control might impact lactational performance. Mutational studies in mice have shown that the deletion of a key mammary super-enhancer [17] and the enhancer driving the *Csn3* gene [17] resulted in reduced lactational performance. Similarly, metabolic genes under STAT5A/B control are also critical for successful lactation [70].

Mechanistic investigations aimed at identifying regulatory elements controlling ‘mammary genes’ have largely focused on *bona fide* milk protein genes, such as *Wap* [15] and *caseins* [16, 17] and the *Stat5a/b* locus itself [44]. Here we show that genes controlling mammary homeostasis are also under STAT5-dependent enhancers. Notably, the *Olah* gene, encoding an enzyme primarily involved in fatty acid metabolism, is abundant in secreting mammary epithelium [71, 72] and under the STAT5B control, likely through a four-partite candidate super-enhancer, similar to that of the casein locus [16, 17]. High OLAH levels have been reported in patients with life-threatening viral disease, including COVID-19 [49], suggesting a role a role viral pathogenesis. While that study did not address the regulation of the *OLAH* gene, our data suggest that its expression in immune cells is controlled by interferons through a super-enhancer, possibly the same used by prolactin in mammary tissue.

In summary, this is the first report to demonstrate that human variants in the JAK-STAT signaling pathway significantly disrupt mammary physiology and lactation. The increasing number of publicly available human genetic databases with associated physiological and medical information such as AllofUs provides an opportunity to investigate the role of genetic variation in lactational diversity in women.

### Limitations of the Study

At this point, the reason for the distorted Mendelian ratio, i.e. the paucity of STAT5B^Y665F^ homozygous mice derived from mating heterozygous females with heterozygous males is not known.

## Electronic Supplementary Material

Below is the link to the electronic supplementary material.

Supplementary Material 1

Supplementary Material 2

Supplementary Material 3

Supplementary Material 4

Supplementary Material 5

Supplementary Material 6

## Data Availability

The ChIP-seq and RNA-seq generated in this study have been deposited in the Gene Expression Omnibus (GEO) database under accession code GSE270652 (reviewer token: epejwmwgblclbof) and GSE270656 (reviewer token: gtopawksfdojner). Other data were obtained from GEO database under accession code GSE115370, GSE145193, GSE161620, GSE115370 and GSE231441.
