# Supplementary material for: Disease-Associated Mutations of the STAT5B SH2 Domain Regulate Cytokine-Driven Enhancer Function and Mammary Development
Source: J Mammary Gland Biol Neoplasia. 2025 Mar 31;30(1):7. doi: 10.1007/s10911-025-09582-8 (PMC11958444; doi:10.1007/s10911-025-09582-8)
Supplement: Supplementary file 1 — Supplementary Material 1 [file 10911_2025_9582_MOESM1_ESM.docx]

**Supplementary information**

**Disease-associated mutations of the STAT5B SH2 domain regulate cytokine-driven enhancer function and mammary development**

Hye Kyung Lee^1^, Jakub Jankowski^1^, Chengyu Liu^2^, Lothar Hennighausen^1^

^1^Section of Genetics and Physiology, National Institute of Diabetes and Digestive and Kidney Diseases, US National Institutes of Health, Bethesda, Maryland 20892, USA.

^2^Transgenic Core, National Heart, Lung, and Blood Institute, US National Institutes of Health, Bethesda, Maryland 20892, USA.

**Supplementary Figures**

**
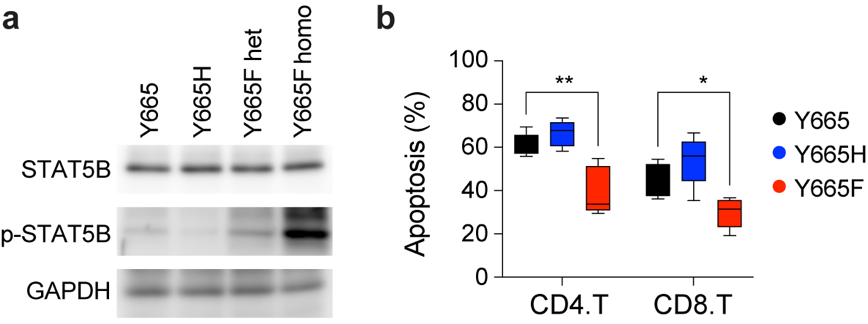
**

**Supplementary Fig. 1 Fig. 1 STAT5B-Y665 mutations in mouse genome.** **A** Expression of wild type and mutant STAT5B proteins. Protein isolated from primary T cells stimulated by cytokines, IL-2 and IL-7, used for western blot analysis. GAPDH was used as a loading control. **B** Apoptosis rate detected by Annexin V and 7-amino-actinomycine staining using flow cytometry (*n* = 5). *P*-value are from one-way ANOVA with Tukey's multiple comparisons test. **P* < 0.05, ***P* < 0.01.

**
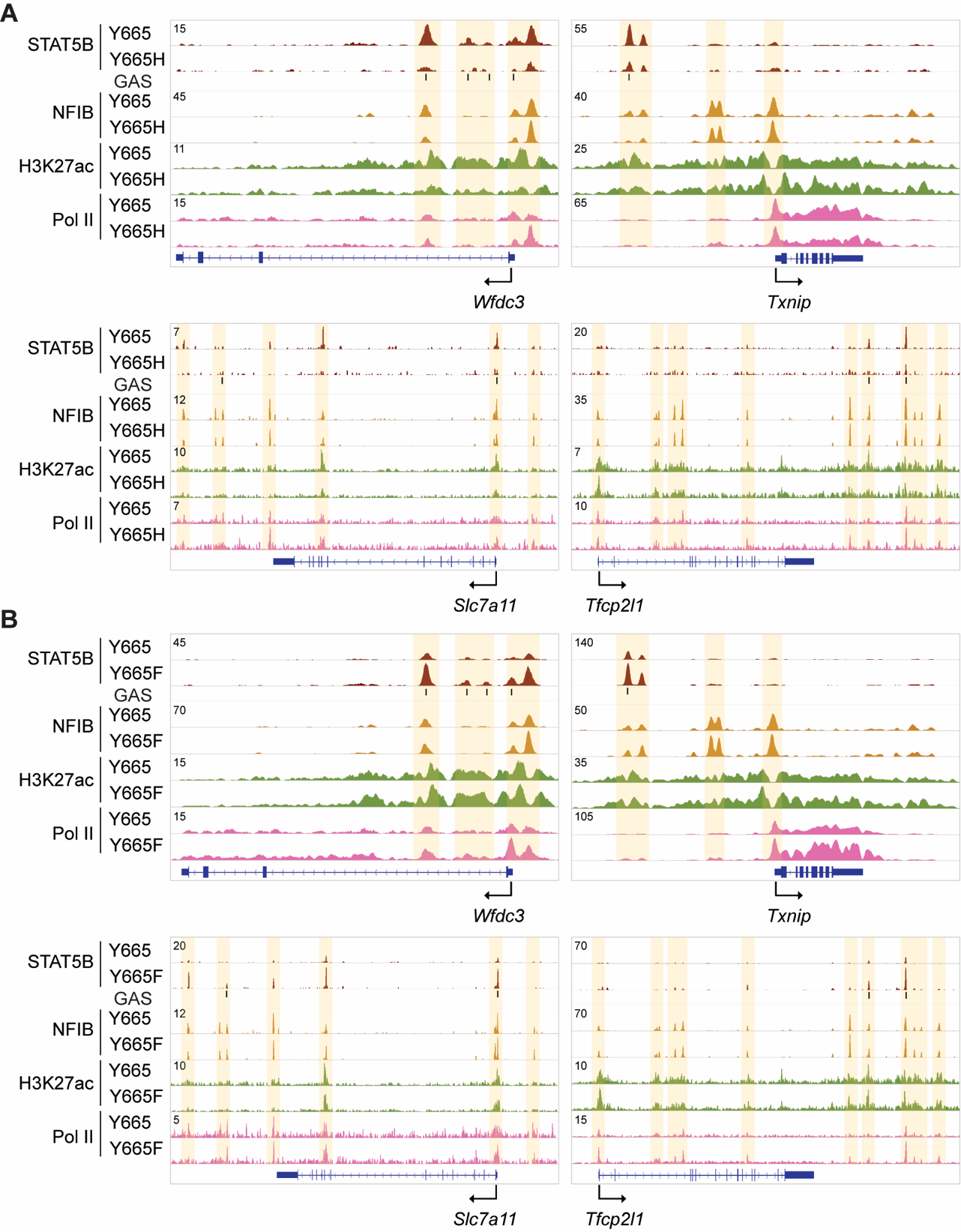
**

**Supplementary Fig. 2. Genomic features of the genes regulated by STAT5B^Y665^ mutations.** Binding of STAT5B and NFIB, H3K27ac, and Pol II at gene loci of skin genes in mammary tissue of wild type (Y665) and *Stat5b^Y665H^* (Y665H) (A) and *Stat5b^Y665F^* (Y665F) (B) mice at day 18.5 of pregnancy. Y665, *n* = 2; Y665H, *n* = 2; Y665F, *n* = 2.

**
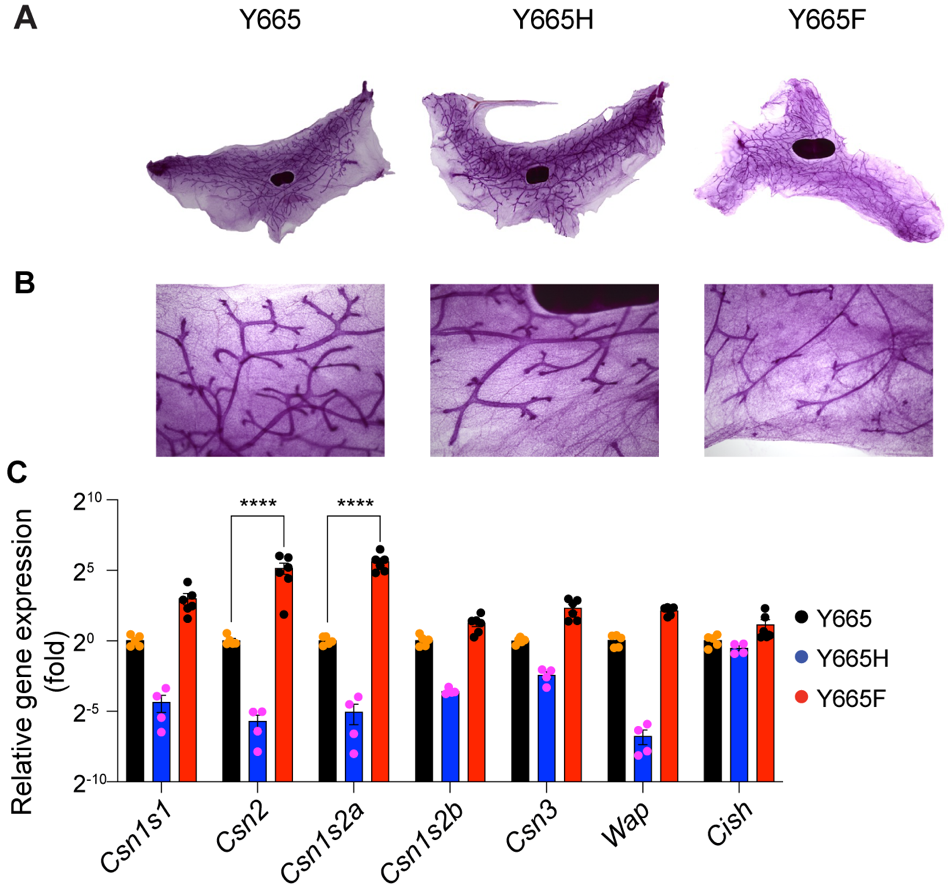
**

**Supplementary Fig. 3 Mammary gland structures in virgin tissue of mutants. A** Image of carmine alum-stained whole mount in virgin mammary gland of WT and mutant mice. Y665, *n* = 4; Y665H, *n* = 2; Y665F, *n* = 9. **B** Zoom-in image of each whole mount image. **C** Expression of representative milk protein genes (*Casein* and *Wap* genes) was measured in mammary tissue of Y665 (WT) and mutant mice collected at virgin by qRT-PCR (Y665, *n* = 5; Y665H, *n* = 4; Y665F, *n* = 6). Results are shown as the means ± SEM of independent biological replicates. *p*-values are from 2-way ANOVA followed by Dunnett’s multiple comparisons test between WT and mutants. *****p* < 0.00001.

**Supplementary Tables**

**Supplementary Table 1.** Sequences of sgRNA for CRISPR/Cas9 and base-editing targeted mice. The donor oligo is contained the desired Y (TAC) to F (TTT) change.

| Target site | sgRNA sequences |
| --- | --- |
| Y665H | 5’-TGAGGTAATTCAGGTCCCCCAGG-3’ |
| Y665F | 5’-TGAGGTAATTCAGGTCCCCCAGG-3’  Donor Oligos  GGAATCTGATGCCTTTTACCACTAGAGACTTCTCTATCCGGTCCCTCGCTGA  CCGCCTGGGGGACCTGAATTTTCTCATATATGTGTTTCCTGATCGGCCAAAG  GATGAAGTATATTCTAAGTACTACACACC |

**Supplementary Table 2.** An overview of the data quality for ChIP-seq and RNA-seq experiments, along with the specificity of antibodies used in ChIP-seq.

**Supplementary Table 3.** List of significantly regulated genes in mammary gland from wild type and Y665H mutant mice at day 18.5 of pregnancy, Fold Change, their *p*-value and adjusted *p*-value.

**Supplementary Table 4.** List of significantly regulated genes in mammary gland from wild type and Y665F mutant mice at day 18.5 of pregnancy, Fold Change, their *p*-value and adjusted *p*-value.

**Supplementary Table 5.** List of significantly regulated genes in mammary gland from wild type and Y665F mutant mice at day 13.5 of pregnancy, Fold Change, their *p*-value and adjusted *p*-value.

**Supplementary Table 6.** List of significantly regulated genes in mammary gland from wild type and Y665H mutant mice at day ten of lactation, Fold Change, their *p*-value and adjusted *p*-value.
